# Environmental Pollutant Anthracene Induces ABA-Dependent Transgenerational Effects on Gemmae Dormancy in *Marchantia polymorpha*

**DOI:** 10.3390/plants13212979

**Published:** 2024-10-25

**Authors:** Juan I. Tolopka, Maya Svriz, Tamara M. Ledesma, Eugenia Lanari, José M. Scervino, Javier E. Moreno

**Affiliations:** 1 Instituto de Agrobiotecnología del Litoral, Centro Científico Tecnológico CONICET Santa Fe, Facultad de Bioquímica y Ciencias Biológicas, Universidad Nacional del Litoral-CONICET, Colectora Ruta Nacional No. 168 km. 0, Paraje El Pozo, Santa Fe 3000, Argentina; juanitolopka@gmail.com (J.I.T.); ledesma_m@live.com.ar (T.M.L.); 2 Instituto de Investigaciones en Biodiversidad y Medioambiente (INIBIOMA), Universidad Nacional del Comahue-CONICET, SC Bariloche, Río Negro 8400, Argentina; mayasvriz@comahue-conicet.gob.ar (M.S.); jmscervino@hotmail.com (J.M.S.)

**Keywords:** polycyclic aromatic hydrocarbons, liverwort tolerance, abscisic acid mutants, gemma germination, regulation, ecological impact

## Abstract

Anthracene, a polycyclic aromatic hydrocarbon (PAH) from fossil fuel combustion, poses significant environmental threats. This study investigates the role of abscisic acid (ABA) in the anthracene tolerance of the liverwort *Marchantia polymorpha* using mutants deficient in ABA perception (Mp*pyl1*) or biosynthesis (Mp*aba1*). In this study, we monitored the role of ABA in the anthracene tolerance response by tracking two ABA-controlled traits: plant growth inhibition and gemmae dormancy. We found that the anthracene-induced inhibition of plant growth is dose-dependent, similar to the growth-inhibiting effect of ABA, but independent of ABA pathways. However, gemmae dormancy was differentially affected by anthracene in ABA-deficient mutants. We found that gemmae from anthracene-exposed WT plants exhibited reduced germination compared to those from mock-treated plants. This suggests that the anthracene exposure of mother plants induces a transgenerational effect, resulting in prolonged dormancy in their asexual propagules. While Mp*pyl1* gemmae retained a dormancy delay when derived from anthracene-exposed thalli, the ABA biosynthesis mutant Mp*aba1* did not display any significant dormancy delay as a consequence of anthracene exposure. These results, together with the strong induction of ABA marker genes upon anthracene treatment, imply that anthracene-induced germination inhibition relies on ABA synthesis in the mother plant, highlighting the critical role of MpABA1 in the tolerance response. These findings reveal a complex interplay between anthracene stress and ABA signaling, where anthracene triggers ABA-mediated responses, influencing reproductive success and highlighting the potential for leveraging genetic and hormonal pathways to enhance plant resilience in contaminated habitats.

## 1. Introduction

Environmental pollution, particularly with compounds that originate from the incomplete combustion of fossil fuels such as polycyclic aromatic hydrocarbons (PAHs), poses a significant threat to agricultural sustainability and ecosystem health [1]. Among these PAHs, anthracene appears as a widespread environmental pollutant to be considered in future scenarios [2]. Approximately 90% of PAHs in the environment are associated with soil particles, and these deposits have been linked to human activities in the industrialized regions of various countries [3]. Specifically, anthracene concentrations in soils range from 0.002 to 0.07 ppm in non-contaminated and aged-PAH-contaminated soils, respectively, with the highest recorded concentration being 100 ppm (≈560 μM) in coal tar [4]. In recent years, important efforts have been made to understand the physiological and biochemical responses of plants to PAH and, more recently, to anthracene exposure [5,6,7,8,9,10]. Notably, several studies on different plant lineages have established a clear inhibitory effect of anthracene on plant growth [5,6,7,8,9]. Moreover, in *Arabidopsis thaliana* (L.) Heynh. and *Marchantia polymorpha* L. subsp. *rudelaris* Bischl. *et* Boissel. Dub. (Tak-1), this inhibition is manifested as a dose-dependent reduction in plant biomass [8,11,12].

In the context of anthracene stress, the plant hormone abscisic acid (ABA) emerges as a critical factor. ABA is a key regulator of plant stress responses, including drought and salinity and the regulation of seed or gemmae dormancy and germination in land plants [13,14,15,16,17]. Comparative analyses with angiosperms using bryophytes like *Physcomitrella patens* and *M. polymorpha* have provided insights into the evolution of land plants and the conservation of ABA core signaling components [17], including the canonical ABA response element present in gene promoter regions [18].

Functional genomics has shown that ABA from bryophytes protects cells from osmotic stress, using signaling components similar to those in angiosperms. For example, the transcription factor ABI3 plays a role in seed desiccation tolerance and dormancy [19]. This factor, conserved across land plants, also contributes to ABA-mediated protonemal desiccation tolerance in *P. patens* [20]. In *M. polymorpha*, ABI3 affects ABA responses in the vegetative thallus and controls gemma dormancy [15]. These similarities in the ABA pathway suggest its existence in a common ancestor of land plants ≈ 450 million years ago [21].

The observed overlap of the transcriptomic response to anthracene or ABA in *M. polymorpha* suggests a potential mechanism wherein plant tolerance to anthracene may involve circuits of the ABA pathways, either through ABA perception or biosynthesis [8]. This similarity in the plant’s response to anthracene and ABA suggests that anthracene exposure may induce ABA synthesis or mimic its effects, indicating a complex interaction between environmental pollutants and the hormonal mechanisms that regulate growth and development in plants.

This study aims to enhance our understanding of plant plasticity in the face of anthropogenic stressors, with a particular focus on the role of the ABA signaling pathway as a mediator of tolerance or susceptibility to PAH toxicity using anthracene as the chemical model compound. Here, we test the interaction between anthracene stress and ABA-controlled traits, monitoring the effects of anthracene on plant growth and gemmae germination, with the latter being relevant as an ABA-regulated reproductive mechanism in liverworts. To explore this hypothesis, we tested how alterations in ABA synthesis and perception pathways influence anthracene tolerance of ABA-related mutants of *M. polymorpha* [13,22]. Our findings demonstrate that gemmae from anthracene-exposed plants exhibit delayed germination, indicative of a transgenerational effect of an ABA-like response, which further supports the hypothesis of ABA synthesis induction or mimetic effects by anthracene [15]. Our results offer a novel perspective on how environmental pollutants can interact with endogenous plant hormonal pathways, influencing key developmental processes and potentially affecting plant fitness and reproduction.

## 2. Results

### 2.1. Plant Growth Inhibition by Anthracene Does Not Rely on ABA Pathways

**Figure 1:**
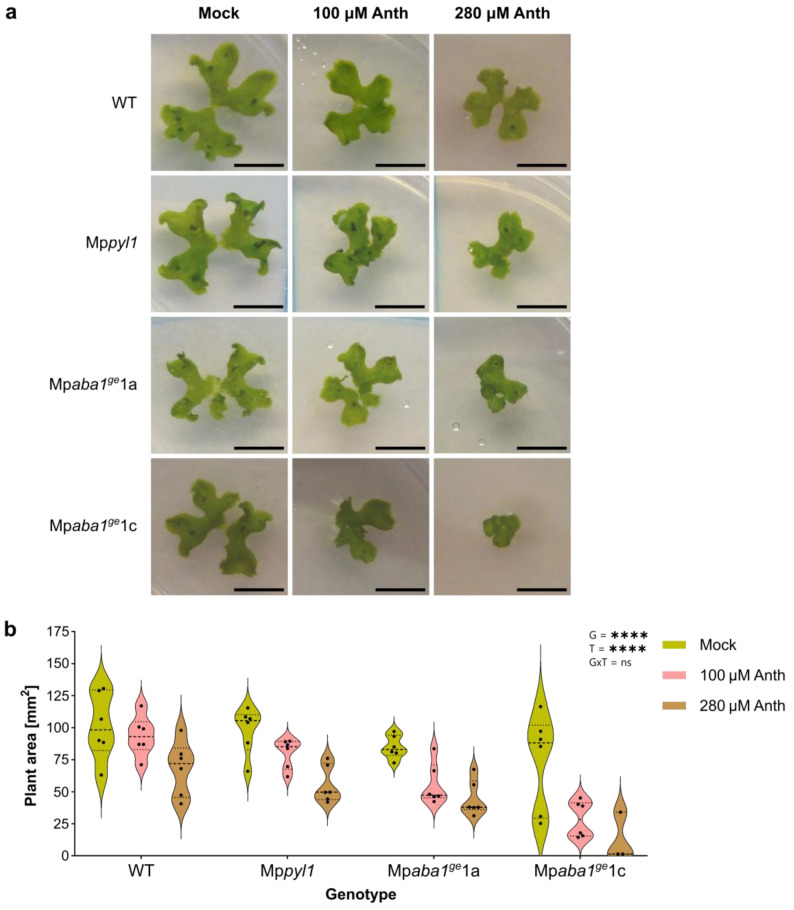
Dose–response inhibition of anthracene on plant area. *M. polymorpha* plants of different genotypes (WT, Mp*pyl1*, Mp*aba1^ge^*1a, and Mp*aba1^ge^*1c) were grown in axenic conditions for 28 days using 0.5X× Gamborg medium supplemented with 100 and 280 μM of anthracene (Anth). Mock plants were used as a control. (**a**) Representative images of 28-day-old plants treated with anthracene, used for area quantification. Scale bars = 1 cm. (**b**) Violin plots show the distribution of individual measurements (n = 6). Each data point is the average of one Petri dish containing four plants. The inset values summarize the statistical significance of the main effects of G and T and the GxT interaction. Asterisks denote significant differences (*p* < 0.001).

Plant growth is inhibited in a dose-dependent manner by anthracene. This was shown before for *A. thaliana* [11] and *M. polymorpha* [8,12]. Since the transcriptional response to anthracene in *M. polymorpha* shares similarities with the ABA response, we proposed that plant tolerance to anthracene could rely, at least in part, on an intact pathway related to ABA perception and/or synthesis [8]. To test this hypothesis, we compared the response of wild-type (WT) *M. polymorpha* plants with mutants impaired in the perception (Mp*pyl1*) or synthesis (Mp*aba1^ge^*1a and Mp*aba1^ge^*1c) of ABA [13,22]. As described before, the plant area of the WT genotype after 4 weeks of growth was progressively inhibited with increasing concentrations of anthracene, with a more pronounced decrease at 280 µM (Figure 1). Similarly, the Mp*pyl1* mutant showed a significant reduction in plant area in a dose-dependent manner (Figure 1b). A comparable effect was observed for the ABA biosynthesis mutant alleles Mp*aba1^ge^*1a and Mp*aba1^ge^*1c (Figure 1b). These results indicate that both genotype and anthracene concentration significantly impact the final plant area (genotype, *p* < 0.001, and anthracene, *p* < 0.001). In other words, the lack of a significant interaction effect (GxT, *p* = 0.329) suggests that the influence of anthracene on plant area does not depend on the genotype, concluding that the changes in plant area due to anthracene concentration affect all genotypes tested similarly.

### 2.2. Role of ABA in Modulating Gemma Germination During Anthracene Exposure

**Figure 2:**
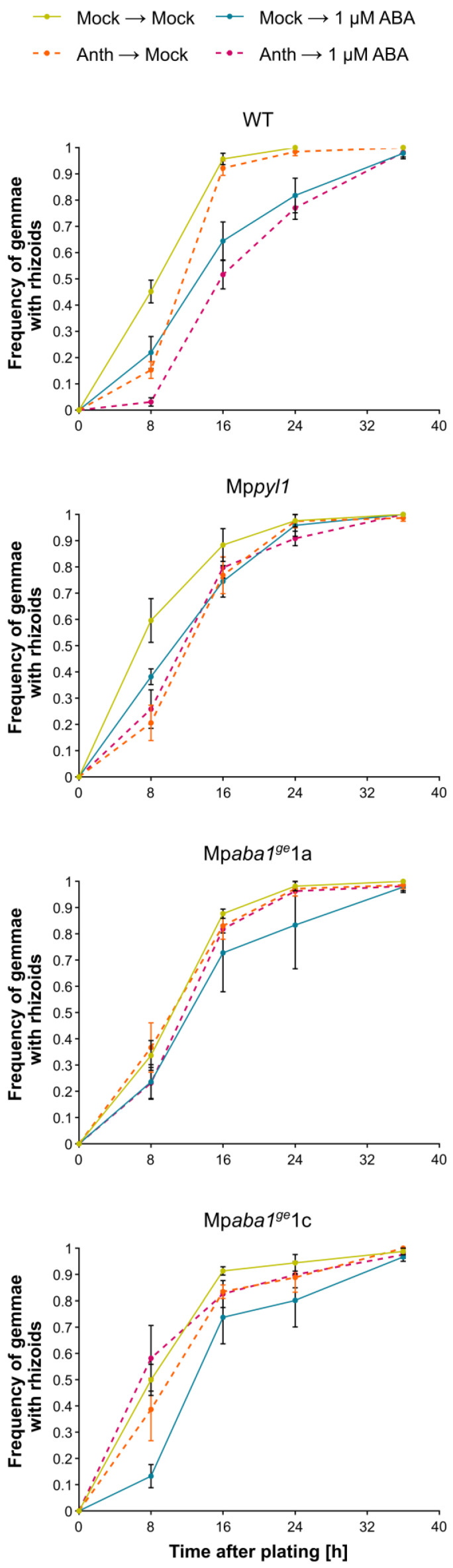
An intact ABA response is required for anthracene to inhibit gemmae germination. Dormant gemmae were placed on 0.5× Gamborg medium supplemented with 0 (Mock) or 1 μM ABA. The gemmae were collected from plants grown under control conditions (Mock) or exposed to 100 μM anthracene (Anth). They were plated on 0.5× Gamborg medium and grown under long-day conditions (80–100 μmol m^−2^ s^−1^) at 22 °C. Rhizoid emergence (germination) was scored at regular intervals of 8 h after plating. Each data point represents the average of three plates, with error bars indicating SE of proportions (n = 3). Each plate has an average of 15 plants. A one-way ANOVA was performed to assess the effect of treatment on the frequency of germination at the 8 h time point. Significant differences were further examined using Fisher’s LSD post hoc test (*p* < 0.05).

To further explore the contribution of the ABA pathway to anthracene tolerance, we also explored gemmae germination, another ABA-controlled trait in liverworts [15]. ABA has been reported to inhibit gemma germination in a dose-dependent manner once it is outside of the gemma cup microenvironment [15]. In addition, it has also been reported that anthracene exposure does not directly affect the gemmae dormancy of *M. polymorpha* and *Lunularia cruciata* (L.) Dumort. ex Lindb [9,12]. We hypothesized that anthracene treatment of the mother plant primes their gemmae for future anthracene exposure, such that gemmae from anthracene-treated plants would display a lower germination frequency compared to gemmae collected from mock-treated plants. Remarkably, this was exactly what happened with WT gemmae (Figure 2). Particularly at early time points, gemmae harvested from mock-treated plants showed increased germination (ca. 40%) compared to those from anthracene-treated plants (ca. 10%, *p*-value: 0.001). A similar inhibitory effect was observed at 8 h for gemmae treated with 1 µM ABA. However, while the anthracene inhibitory effect lasted until the end of the kinetic response, the ABA-induced dormancy effect had already faded after 16 h, reaching the maximum germination frequency. As expected, the combined treatment of anthracene + ABA superimposed a stronger inhibition of gemmae germination, lasting to the end of the experiment (Figure 2). For the mutant plants, the germination frequency of Mp*pyl1* at 8 h was significantly higher than the germination of WT gemmae (60% vs. 45%, Figure 2), showcasing the release of the dormancy due to the lack of a functional MpPYL1. Even more, upon 8 h of ABA stress, the germination frequency of Mp*pyl1* was higher than WT gemmae germination, highlighting the physiological role in ABA perception by MpPYL1 during gemmae dormancy. More importantly, at early time points (8 and 16 h), gemmae from mock plants of the Mp*pyl1* mutant germinated significantly faster than gemmae from the other three ABA-inducing treatments (ABA, anthracene, and ABA + anthracene, *p*-value: 0.014) (Figure 2). This result suggests that the MpPYL1 ABA receptor is required for a full response to ABA, while the contribution of other MpPYL receptors (MpPYL2-5) to anthracene tolerance should be further explored using higher-order mutants. Notably, the Mp*aba1^ge^*1a and Mp*aba1^ge^*1c germination response exhibited apparent differences with the WT when coming from mother plants exposed to anthracene. In this regard, the germination of the Mp*aba1^ge^*1a and Mp*aba1^ge^*1c gemmae showed a mild inhibition response to exogenous ABA, and more importantly, with their germination not further affected by the anthracene exposure of their mother plants (*p*-value: 0.458). This result indicates that the anthracene-induced inhibition of germination requires ABA synthesis from the mother plant.

**Figure 3:**
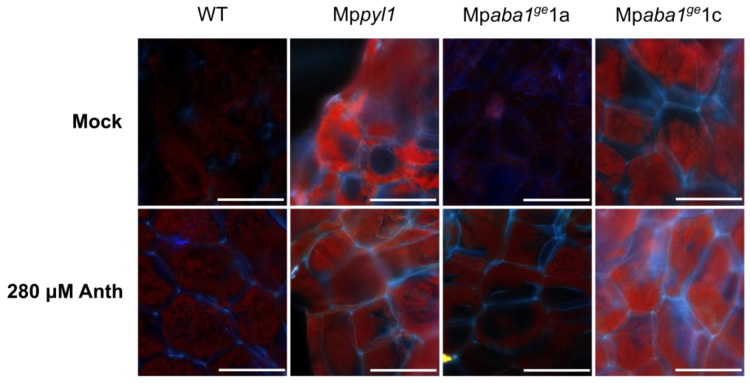
Quantification of autofluorescence associated with anthracene internalization in plants. *M. polymorpha* plants of different genotypes (WT, Mp*pyl1*, Mp*aba1^ge^*1a, and Mp*aba1^ge^*1c) were grown in 0.5× Gamborg medium supplemented or not with 280 μM of anthracene (Anth) for 5 d. Mock-treated plants were used as a control. Representative pictures of autofluorescence are shown here (scale bars = 20 μm).

To rule out the chance that differences in the anthracene tolerance of ABA mutants were related to different internalization rates of the pollutant, we quantified the intracellular concentration of anthracene using epifluorescence microscopy. As shown in Table 1 and Table S1, anthracene internalization within plant cells was already detected 5 d after the initiation of the experiment (Figure 3). This internalization increased over time. Whereas the WT, Mp*aba1^ge^*1a, and Mp*aba1^ge^*1c genotypes presented similar absolute fluorescence intensities, Mp*pyl1* showed higher intensity in both the mock and 280 µM treatments. This enhanced autofluorescence of the Mp*pyl1* mutant may be due to the constitutive presence of autofluorescent compounds in this mutant. Interestingly, we found consistent anthracene measurements across genotypes, suggesting that the anthracene internalization process is not responsible for the observed differences.

Taken together, these results suggest that an intact ABA biosynthetic pathway, mediated by MpABA1, but not the ABA perception controlled by MpPYL1, is required for mounting a full tolerance response to the anthracene of *M. polymorpha* plants.

### 2.3. ABA Response Marker Genes Are Induced by Anthracene

**Figure 4:**
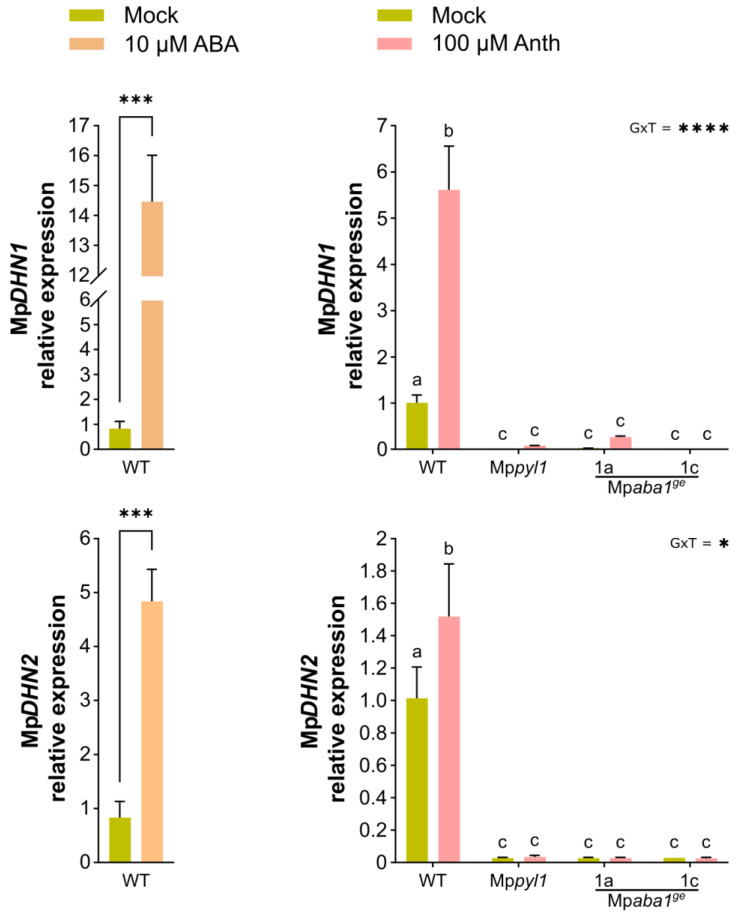
Anthracene induction of ABA-responsive genes is abrogated in ABA-related mutants. Effect of 100 μM anthracene (Anth) on the induction of ABA-responsive genes 24 h after elicitation: Mp*DHN1* and Mp*DHN2*. WT plants treated with 10 μM ABA for 24 h were used as a positive control. Asterisks denote significant differences between treatment means (*p* < 0.001). Transcript abundance was measured as the response variable after chemical treatment and expressed relative to the level detected in mock WT plants. Thin bars indicate 1 SEM (n = 3 replicates). The GxT interaction is significant in both panels (*p* < 0.05); different letters indicate significant differences between treatment means.

Exogenous ABA application, as well as the anthracene treatment of *M. polymorpha* plants, induces the expression of *DEHYDRIN 1* (Mp*DHN1*) and *DEHYDRIN 2* (Mp*DHN2*), two ABA marker genes of *M. polymorpha* [8,15,23]. We wondered whether their expression would be differentially altered in the ABA mutant plants following anthracene treatment. Through the qPCR analysis of 30-day-old plants treated with 100 µM anthracene, we found that both ABA or anthracene upregulated *MpDHN1* and *MpDHN2* expression in WT plants (Figure 4). Interestingly, both the ABA synthesis and ABA perception mutants showed no induction of Mp*DHN1* and Mp*DHN2* in anthracene-exposed plants. These results confirm that ABA synthesis and perception are activated upon anthracene treatment and support the fact that the ABA circuit is required for the anthracene tolerance response of *M. polymorpha*.

## 3. Discussion

Studies on the impact of polycyclic aromatic hydrocarbons (PAHs), specifically anthracene, on the growth and development of *M. polymorpha* and *L. cruciata* are providing valuable insights into liverwort responses to environmental pollutants. This study supports previous findings that anthracene stress leads to a significant reduction in plant biomass in a dose-dependent manner [8,11,12]. This growth-inhibition response to anthracene is conserved across major land plant lineages, including both vascular and non-vascular plants, highlighting the robust and pervasive nature of anthracene toxicity to plant systems [13,22]. Interestingly, our results also demonstrate that while both vascular and non-vascular plants exhibit similar responses to anthracene contamination, vascular plants like *A. thaliana* are more sensitive to higher concentrations. Specifically, anthracene concentrations higher than 100 µM were lethal to *A. thaliana*, whereas *M. polymorpha* still tolerates 280 µM and 560 µM anthracene [12] (this study). This information is relevant for understanding the impact on plant communities growing in anthracene-polluted environments, as it suggests that species composition and ecosystem resilience may shift depending on the differential tolerance levels observed between vascular and non-vascular plants.

Although the impact of anthracene on the growth of the mutants was evident, their tolerance was similar to the WT plant. This result suggests that while ABA pathways contribute to the plant’s ability to cope with stress, they do not regulate the plant growth inhibition response induced by anthracene. This outcome aligns with observations in other environmental stress contexts, where plants employ a multiplicity of signaling pathways and molecular mechanisms to mitigate stress effects, suggesting a complex network of responses beyond ABA pathways [24]. It is plausible that other stress-related phytohormones, such as ethylene [25,26] or jasmonic acid [27], and other stress-related metabolites with specific roles to various stressors, might also contribute to modulating the plant growth effects of anthracene exposure. Moreover, the observed anthracene-induced growth inhibition, without significant genotype-specific differences, underscores the importance of identifying other genetic and epigenetic factors that could influence liverwort resilience to anthracene stress. Since the *M. polymorpha* genome encodes for five MpPYL receptors (MpPYL1-5), we cannot rule out that other MpPYL receptors regulate the plant growth inhibition response mediated by anthracene. In this regard, it would be interesting to test the anthracene response of a higher-order mutant of MpPYL receptors.

Dormancy plays a crucial role in the ecophysiology of land plants, influencing species fitness and population dynamics. The dormancy response integrates various internal and external cues, with ABA playing a major role as an internal factor [15,22] and anthracene as an external factor examined in this study. Anthracene does not affect the gemmae dormancy of *M. polymorpha* [12] or *L. cruciata* [9], likely because anthracene internalization (Table 1, differences observed at day 5—a daily scale) takes longer than dormancy break (Figure 2, differences observed at 8 h—an hourly scale). Altering these internal and external factors can affect both gemmae dormancy and germination. In line with the similar transcriptional responses observed for ABA- and anthracene-treated plants [8], we found that gemmae from anthracene-exposed plants exhibited extended dormancy, comparable to the level registered with ABA treatment (Figure 2). These results suggest that dormancy by anthracene exposure might also involve ABA pathways. In addition, the gemmae of Mp*pyl1* mutant plants exhibited a weaker dormancy than gemmae from WT plants, even in response to ABA (Figure 2). This observation is consistent with previous findings reported in *M. polymorpha* [28] and in *A. thaliana*, where the duodecuple mutant of AtPYL ABA receptors exhibited a strong insensitivity of seed germination in response to exogenous ABA treatment [29].

The parallel between anthracene effects and ABA-mediated dormancy underscores the intricacies of plant–pollutant interactions and reveals potential adaptive strategies that plants might employ in anthracene-laden environments. More importantly, our results also support a transgenerational effect of anthracene on gemmae dormancy. The extended dormancy frequencies observed in gemmae from anthracene-exposed plants underscore the potential for anthracene to activate ABA pathways that regulate growth and development in asexual propagules [15]. This finding is particularly intriguing as it suggests a novel avenue through which environmental pollutants can influence plant physiological processes. It also raises questions about the ecological and evolutionary implications of such interactions, especially in terms of reproductive success and population dynamics in contaminated environments. In light of these results, it will be necessary to further explore two different levels of responses that interact to determine the plant establishment success in the field, with the advantage that these are testable hypotheses. First, evaluate gemmae establishment success on soil, either coming from plants exposed or not to anthracene and growing them on soil with different levels of anthracene. Second, use novel mutants of the ABA pathway to understand their contribution to gemma establishment. These investigations will provide a better assessment of the ecological relevance of liverwort resilience mechanisms to the plant community in contaminated environments and identify targets for enhancing plant tolerance to anthropogenic pollutants.

## 4. Materials and Methods

### 4.1. Plant Materials and Growth Conditions

The *Marchantia polymorpha* L. subsp*. rudelaris* Bischl. et Boissel. Dub. plants used in this study are from a Japanese male accession (Tak-1). For maintenance, plants were grown on Petri dishes containing agar solidified (1%) 0.5× Gamborg’s B5 medium, pH 5.5, in growth chambers with a 16/8 h photoperiod (60–70 μmol m^−2^ s^−1^) at 22 °C. Plants were maintained under axenic conditions to collect spontaneously produced gemmae from gemma cups. When necessary, gemmae were collected from plants grown with the same media supplemented with 100 µM anthracene, which still allowed for natural gemmae production. Gemmae were randomly collected from different gemma cups across various plants, without regard for gemmae size or age. The mutant Mp*pyl1^ge2b^*, referred to as Mp*pyl1* throughout the manuscript, is a deficient allele of the ABA receptor MpPYL1 [13], whereas the Mp*aba1^ge^*1a and Mp*aba1^ge^*1c are two different alleles of MpABA1 that show impaired ABA synthesis [22]. We explored the physiological responses of male accessions, as all genome-edited lines were generated in a Tak-1 background using the thallus-transformation protocol [30]. As previously reported by our group, male and female accessions of *M. polymorpha* may respond to similar environmental signals with varying intensity but consistently in the same direction [23].

### 4.2. Anthracene Treatments and Plant Area Measurement

Based on growth curves for *M. polymorpha* in a previous study [8,12], the culture medium was supplemented with anthracene (95% purity, cat. number 31581, Sigma-Aldrich, St. Louis, MO, USA) to achieve working concentrations of 100 µM and 280 µM. This study established a dose-dependent effect for anthracene on thallus growth for these working concentrations, also showing that *M. polymorpha* tolerates concentrations of up to 560 µM of anthracene [8]. To prevent the toxic effects of acetone (catalog # 702110, Cicarrelli, San Lorenzo, Santa Fe, Argentina), the media was stirred at 50 °C for 20 min to allow acetone evaporation. As a control for toxicity, the same volume of acetone was added to the mock treatment and evaporated in the same way. Each treatment was replicated a minimum of three times.

Four gemmae were transferred onto Petri dishes containing either the anthracene-supplemented medium or the control (mock) medium. The experiment was conducted over 30 d. Pictures of the plants were taken on day 1 and weekly after that until the end of the experiment. Plant area quantification was performed using ImageJ v1.52p (accessed on 4 August 2019 or later) [31]. At the end of the experiment, plants were harvested and immediately preserved in liquid nitrogen for subsequent qRT-PCR experiments.

### 4.3. Gemma Dormancy Scoring

Gemma dormancy was assessed by the presence or absence of visible rhizoids [15]. Gemmae with visible rhizoids were considered non-dormant, while gemmae lacking visible rhizoids were scored as dormant. This analysis was conducted on gemmae from 4-week-old plants grown under the specified conditions. For each treatment, 15 gemmae per genotype were transferred onto Petri dishes, with three replicates per treatment. ABA was prepared as a 0.1 M stock solution in 99.5% ethanol and stored at −20 °C. The ABA solution was added to the medium, with a mock treatment using ethanol alone.

### 4.4. Anthracene Autofluorescence in Plant Tissues

The timing and extent of anthracene uptake by the four genotypes were assessed in a time-course experiment. Gemmae from each genotype were transferred to Petri dishes and exposed to mock and 280 µM treatments under similar conditions as in previous trials. A sufficient number of gemmae were planted to ensure the collection of individuals at each sampling point, set at 5, 10, and 30 d. To examine anthracene internalization within the thallus of each individual, thallus fragments were collected and rinsed with distilled water to eliminate the residual anthracene from the plant surface. These fragments were then observed under an epifluorescence microscope (Olympus Optical Co., Tokyo, Japan) using excitation and emission wavelengths of 365 nm and 420 nm, respectively [12,32]. Images (12-bit resolution) were captured using a Leica DFC425 C CCD camera. Fluorescence intensity in the blue channel was measured using ImageJ v1.52p (accessed on 4 August 2019 or later) [31]. Peak histogram values were obtained by measuring fluorescence from 30 randomly selected circular regions on the cell walls, with results expressed as fluorescence intensity in absorbance units (A.U.).

### 4.5. RNA Extraction and RT-qPCR

Total RNA was extracted from plant tissues using TRI Reagent^®^ (MRC) according to the manufacturer’s instructions, followed by LiCl precipitation. RNA quantity and purity were assessed using a NanoDrop 2000 Spectrophotometer (ThermoFisher, Waltham, MA, USA). cDNA synthesis was carried out in 15 μL reactions with RevertAid Reverse Transcriptase (ThermoFisher, Waltham, MA, USA.) and oligodT primers at 42 °C. Quantitative RT-PCR (RT-qPCR) was performed using SsoAdvanced™ Universal SYBR^®^ Green Supermix (Bio-Rad, Hercules, CA, USA) on a CFX Connect Real-Time PCR Detection System (Bio-Rad). The primers used in the qRT-PCR are MpACT_Fw AGGCATCTGGTATCCACGAG, MpACT_Rv ACATGGTCGTTCCTCCAGAC, MpDHN1_Fw GGGCCCTCTACTCCTGGTTA, MpDHN1_Rv TTAGCATCATACGGGGTGGC, MpDHN2_Fw GATAAGCTCACGGGACACGA, and MpDHN2_Rv CTTCGGAGACTTGGGCGAAT. MpACT was used for normalization according to the ΔΔCt method. Three biological replicates, tested by duplicates, were used to calculate the standard deviation. Each replicate was obtained by pooling tissue from 3 to 4 individual plants.

### 4.6. Statistical Analysis

Statistical analyses were conducted using Prism v9.4.1 (GraphPad Software, San Diego, CA, USA). Changes in plant area, gemmae dormancy, and gene relative expression levels were analyzed using either ANOVA or *t*-tests, as appropriate. Fisher’s LSD method was used for pairwise comparisons. Graphics of all the data were also generated with this software. A linear model was used to analyze the fluorescence intensity by treatments, time, and genotype. Multiple comparisons of means were made using Tukey post hoc contrasts *p* < 0.05.

## 5. Conclusions

This study describes a transgenerational effect of anthracene on liverworts, particularly in relation to gemmae dormancy. Our findings reveal that anthracene exposure extends dormancy through the ABA biosynthesis pathway. This discovery suggests a previously unexplored mechanism through which pollutants like anthracene can impact plant physiological processes across generations.

The ability of anthracene to induce extended dormancy in asexual propagules underscores its capacity to influence plant establishment, likely affecting reproductive success and population dynamics in contaminated environments. This could lead to shifts in species fitness and adaptation strategies in polluted habitats, potentially affecting plant communities.

These findings open a new avenue of research into how environmental pollutants interact with plant hormonal pathways and suggest the need for further studies to understand the broader network of stress-related hormones involved in the anthracene tolerance response of land plants.

## Figures and Tables

**Table 1:** Progress of anthracene internalization in plant tissues. Mean values (±SE) of fluorescence intensity (A.U: absorbance units) of anthracene present in *M. polymorpha* plants of different genotypes (WT, Mp*pyl1*, Mp*aba1^ge^*1a, and Mp*aba1^ge^*1c). Plants grown under axenic conditions were harvested at different times. The 0.5× Gamborg medium was supplemented either with 280 μM of anthracene or with no anthracene used as a control (Mock). Asterisk (*) indicates significant differences between the mock and 280 µM treatments. Different capital letters indicate significant differences between the fluorescence intensity at different times for the 280 µM concentration within each genotype. Different lowercase letters indicate significant differences in fluorescence intensity between genotypes for each treatment and time point. Tukey post hoc contrasts *p* < 0.05.

Genotype	Autofluorescence Units Average (A.U)					
	5 Days		10 Days		30 Days	
	Mock	280 µM	Mock	280 µM	Mock	280 µM
WT	64.591 ± 3.121 b	80.508 ± 3.955 Ab*	59.941 ± 2.675 b	99.704 ± 3.647 Bb*	58.720 ± 2.622 b	120.650 ± 5.291 Cb*
Mp*pyl1*	76.148 ± 2.078 a	110.864 ± 4.440 Aa*	79.576 ± 3.643 a	126.433 ± 6.243 Ba*	79.705 ± 3.783 a	163.023 ± 5.796 Ca*
Mp*aba1^ge^*1a	53.191 ± 2.827 b	74.537 ± 2.946 Ab*	50.775 ± 1.713 b	110.239 ± 5.186 Bb*	63.132 ± 2.560 b	135.118 ± 6.202 Cb*
Mp*aba1^ge^*1c	39.878 ± 2.385 b	68.543 ± 2.398 Ab*	44.166 ± 2.308 b	65.538 ± 2.295 ABb*	53.336 ± 2.237 b	88.426 ± 3.907 Cb*

## Data Availability

Data are contained within the article and Supplementary Materials.

## Supplementary Materials

The following supporting information can be downloaded at: https://www.mdpi.com/article/10.3390/plants13212979/s1, Figure S1: Dose–response impact of anthracene on plant area over 4 weeks, Table S1: Statistical results performed on fluorescence intensity of gemmae.
