# Supplementary material for: Environmental Pollutant Anthracene Induces ABA-Dependent Transgenerational Effects on Gemmae Dormancy in Marchantia polymorpha
Source: Plants (Basel). 2024 Oct 25;13(21):2979. doi: 10.3390/plants13212979 (PMC11548294; doi:10.3390/plants13212979)
Supplement: Supplementary file 1 [file plants-13-02979-s001.zip › plants-3248848-supplementary.pdf]

Supplementary Information

Environmental pollutant anthracene induces ABA-dependent transgenerational effects on gemmae dormancy in *Marchantia polymorpha*

Juan I. Tolopka[1†], Maya Svriz[2†], Tamara M. Ledesma[1], Eugenia Lanari[2], José M. Scervino[2], Javier E. Moreno[1,*]

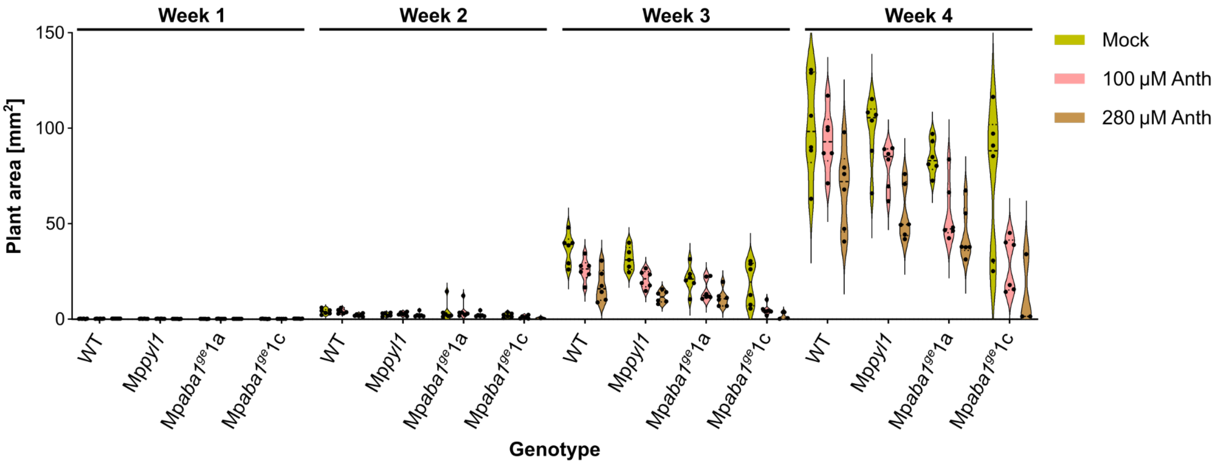

Figure S1: Dose-response impact of anthracene on plant area over 4 weeks. *M. polymorpha* plants of different genotypes (WT, Mp*pyl1*, Mp*aba1*^{ge}1a and Mp*aba1*^{ge}1c) were grown in axenic conditions using 0.5X Gamborg medium supplemented with 0 (Mock), 100 and 280 µM of anthracene (Anth). Violin plots show the distribution of individual measurements (n = 6). Each data point is the average of one Petri dish containing four plants. The inset values show a summary of the statistical significance of the main effects of G and T, and the GxT interaction. Asterisks denote significant differences ($p < 0.001$).

Fluorescence intensity	*df*	*F*	*P*
Treatments	1	598.886	<0.001
Time	2	92.731	<0.001
Genotype	3	130.600	<0.001
Treatments x time	2	42.028	<0.001
Treatments x genotype	3	9.221	<0.001
Time x genotype	6	4.657	<0.001
Treatments x time x genotype	6	3.640	<0.001
Residuals	694		

Table S1: Statistical results performed on fluorescence intensity of gemmae. Degrees of freedom (df), Fisher's F statistics (F) and error probabilities (P) are presented for each factor and their interactions from Table 1. Fluorescence intensity as the response variable, with treatments, time, and genotypes as factors.
